# Maternal-Autoantibody-Related (MAR) Autism: Identifying Neuronal Antigens and Approaching Prospects for Intervention

**DOI:** 10.3390/jcm9082564

**Published:** 2020-08-07

**Authors:** Katya Marks, Ester Coutinho, Angela Vincent

**Affiliations:** 1 Medical Sciences Division, John Radcliffe Hospital, University of Oxford, OX3 9DU Oxford, UK; katya.marks@ccc.ox.ac.uk; 2 Department of Basic and Clinical Neuroscience, Institute of Psychiatry, Psychology and Neuroscience, Maurice Wohl Clinical Neuroscience Institute, King’s College London, SE5 9RT London, UK; ester.coutinho@kcl.ac.uk; 3 Medical Research Council Centre for Neurodevelopmental Disorders, King’s College London, SE1 1UL London, UK; 4 Nuffield Department of Clinical Neurosciences and Weatherall Institute for Molecular Medicine, University of Oxford, OX3 9DS Oxford, UK

**Keywords:** autism, neurodevelopmental disorder, autoantibodies, autoimmunity, pregnancy, placental antibody transfer, CASPR2, NMDAR, immunotherapy

## Abstract

Recent studies indicate the existence of a maternal-autoantibody-related subtype of autism spectrum disorder (ASD). To date, a large number of studies have focused on describing patterns of brain-reactive serum antibodies in maternal-autoantibody-related (MAR) autism and some have described attempts to define the antigenic targets. This article describes evidence on MAR autism and the various autoantibodies that have been implicated. Among other possibilities, antibodies to neuronal surface protein Contactin Associated Protein 2 (CASPR2) have been found more frequently in mothers of children with neurodevelopmental disorders or autism, and two independent experimental studies have shown pathogenicity in mice. The N-methyl-D-aspartate receptor (NMDAR) is another possible target for maternal antibodies as demonstrated in mice. Here, we discuss the growing evidence, discuss issues regarding biomarker definition, and summarise the therapeutic approaches that might be used to reduce or prevent the transfer of pathogenic maternal antibodies.

## 1. Introduction

Autism spectrum disorder (ASD) is a lifelong developmental disability with an estimated global prevalence of 0.6–1.5% [1]. It is characterised by problems with communication, social interaction and restrictive, repetitive activities and interests (RRBIs) [2]. While diagnosis is still informed by these behavioural symptoms alone, recent decades have seen considerable advances in our understanding of the aetiology of ASD, with investigations into the contributions made by genetics [3], the environment [4] and the immune system [5]. Since 1971, there has been growing evidence of the association between family history of auto-immune disease and risk of autism [6,7]. Concordantly, certain major histocompatibility complex (MHC) haplotypes and polymorphisms have been identified as autism risk genes [8,9]. Early research in this area was closely followed by several critical studies in the description of maternal-autoantibody-related (MAR) autism, a sub-type of ASD in which autoantibodies reactive to foetal brain proteins are present in the maternal serum (Table A1 and Table A2).

Table A1 and Table A2 provide a comprehensive summary of the studies on MAR autism to date. A combination of associative human studies and passive transfer and immunisation studies in animals provides convincing proof of the concept of MAR autism. Furthermore, there is increasing data on the antibodies potentially involved. This article will discuss maternal autoantibodies targeting Contactin Associated Protein 2 (CASPR2), the N-methyl-D-aspartate receptor (NMDAR) and a selection of intracellular antigens, with particular focus on the pathogenic potential of each antibody. We will further speculate on mechanisms of autoantibody generation. This is still an emerging field and predictions are premature, but MAR autism could represent a relatively small but potentially preventable proportion of all patients.

Looking forward, it is tempting to imagine a prophylactic treatment for MAR autism. Indeed, various treatments established for other autoimmune disorders that are transmitted to the developing foetus might also be effective here (Table A3). However, a number of factors complicate the use of such treatments. Crucially, the ethics of administering treatment to a healthy mother and her foetus for prevention of a developmental disorder are questionable given our current inability to predict with accuracy the case-by-case outcome of maternal autoantibodies. Therefore, this article concludes by considering potential treatment approaches to MAR autism, together with the issues involved in realising a feasible and optimal strategy. These include (1) defining specific MAR autism biomarkers, (2) understanding the contribution of other variables to foetal prognosis in the presence of pathogenic autoantibodies and (3) determining the appropriate window in gestation for treatment.

## 2. Early Studies

Table A1 describes 13 studies that have associated patterns of maternal serum reactivity with an outcome of ASD or other neurodevelopmental disorder in the child. The first study to indicate a possible association between maternal antibodies and autism was carried out by Warren et al. (1990) [10], who demonstrated that plasma from six of eleven mothers of an autistic child had antibodies reactive to lymphocytes of the child. Considering the substantial cross-reactivity between lymphocyte and neuronal antigens, this study prompted further investigation into the concept of MAR autism.

In the mid-1990s came two case studies on autoantibody-related neurodevelopmental disorder. Barnes et al. (1995) [11] described a mother whose four pregnancies were affected by foetal arthrogryposis multiplex congenita (AMC), a rare condition characterised by multiple joint contractures, caused by lack of foetal movement in utero, and previously associated with maternal myasthenia gravis (MG). The mother had a history of shoulder and facial muscle weakness, but MG was not diagnosed until after her fourth pregnancy, when she had highly positive serum acetylcholine receptor (AChR) antibodies. Vincent et al. (1995) [12] described a second mother who, despite remaining healthy herself, had six consecutive pregnancies affected by AMC. Serum taken before the sixth pregnancy contained a high titre of AChR antibodies. Foetal movements were initially normal until 15–18 weeks, but repeated plasma exchange from 10 weeks gestation prolonged foetal movements, indicating that onset/progression of AMC could be delayed. This implicated maternal factors over a genetic cause in the pathogenesis. Furthermore, a maternal-autoantibody-mediated cause of AMC was heavily suggested by the recurrence of AMC in all pregnancies (both studies) and the fact that foetal movements were observed until 15–18 weeks, the time at which foetal immunoglobulin G (IgG) levels begin to rise rapidly. This hypothesis was strengthened by demonstration that the maternal serum antibodies selectively inhibited the foetal form of AChR (Riemersma et al., 1997) [13], making this the first study to implicate clearly maternal autoantibodies as a cause of a neurodevelopmental disorder and catalysing further research into this novel field. Witebsky’s postulates for antibody-mediated disorders, as modified by Rose and Bona [14], rely on transfer of disease to an experimental model (as well as transfer of disease from mother to her offspring). To establish an animal model, Jacobson et al. (1999) [15] injected pregnant mouse dams with plasma from four AChR antibody-positive women whose foetuses were affected with severe AMC. Many of the foetuses displayed fixed joint contractures and other deformities resembling AMC. This passive transfer animal model has since been used repeatedly to study the effects of ASD-salient maternal antibodies on foetal development (Table A2).

The first such study looking at autism was done by Dalton et al. (2003) [16] who injected pregnant mouse dams with plasma from a mother who had three surviving children: One typically developing, one with autism and one with a specific language disorder. The mother’s serum contained antibodies that bound to rodent Purkinje cells and to the surface of a neuroblastoma cell line, suggesting a potentially pathogenic neuronal antibody. Compared to controls, the mouse offspring displayed a delayed righting reflex, decreased exploration and decreased ability to balance on the multiple static rods test. While these behavioural measures were far from specific to autism, the reduced exploratory behaviour echoed the characteristic RRBIs seen in autism, and the reduced balancing and changed cerebellar metabolites indicated possible cerebellar dysfunction which has been implicated in the pathophysiology of autism [17]. A strength of this study was its tightly controlled diagnostic criteria for psychiatric disorder; their diagnoses were based on independent clinical assessments by three paediatric specialists. Thus, Dalton et al. were the first to provide causative evidence that maternal factor can induce neurodevelopmental disorder, and with particular direction towards autism.

The story of MAR autism grew significantly from 2007 onwards when a number of studies linked autism with maternal serum reactivity [18,19,20,21]. Western blots of mammalian brain extracts were used as the method of antibody detection in these studies and protein bands were identified by maternal serum IgG in cases but not in controls. Others studied the reactivity of plasma from mothers of an autistic child using mouse brain sections and observed an increased prevalence of anti-brain reactivity in the 10.5% of 2431 cases compared with 2.6% of 653 controls [22]. While the plasma samples used were not mid-gestational, these studies support the existence of MAR autism. However, the method used did not identify specific antigenic targets or identify whether they were intracellular rather than membrane proteins.

Table A2 lists several passive transfer animal studies that have aimed at establishing causative evidence for the association between autism and maternal serum reactivity. Martin et al. (2008) [18] advanced on previous work with the first IgG passive transfer model, in which purified pooled IgG, rather than whole plasma, from mothers of autistic children produced stereotypies and hyperactivity in rhesus macaques. The demonstration of these changes in a primate model makes this particularly compelling evidence. A limitation common to most of the studies listed in Table A1 and Table A2, however, was the use of maternal plasma samples collected several years after the children’s birth [10,16,18,19,20,21,22,23,24,25,26,27,28,29], by which time maternal antibody profiles might have changed. Nevertheless, with repetition across studies and in large cohorts the overall significance of this limitation is reduced. Furthermore, a study by Croen et al. was successful in confirming mid-gestational presence of antibodies to an autism-specific pattern of intracellular antigens [20].

**Figure 1:**
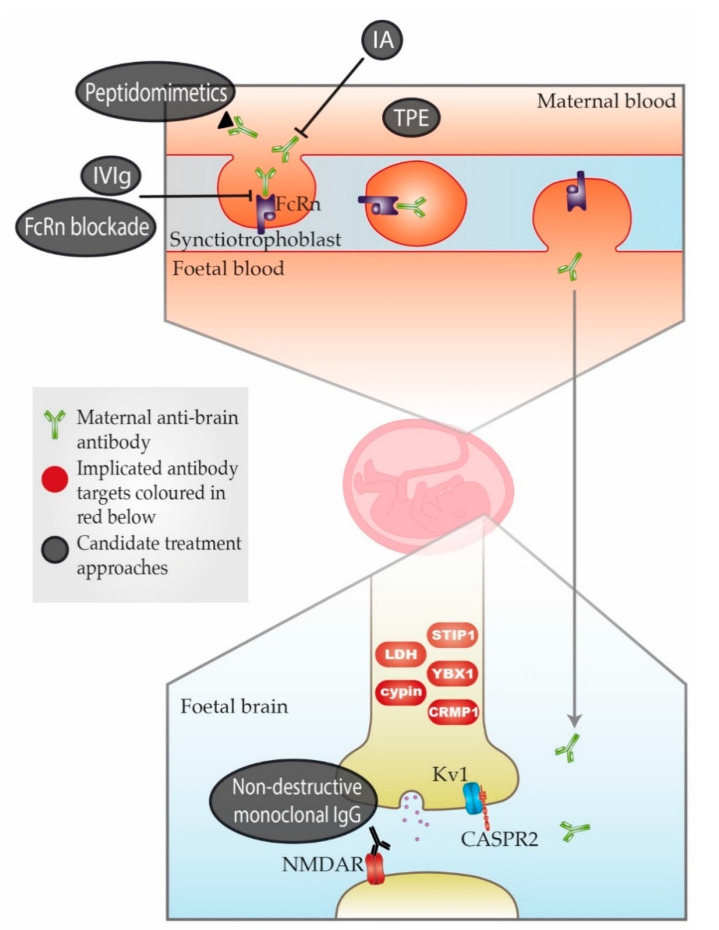
Schematic representation of maternal-autoantibody-related (MAR) autism pathogenesis and potential therapeutic approaches. Placental antibody transfer is mediated by the neonatal Fc receptor (FcRn). Thus, pathogenic maternal antibodies cross into foetal circulation and reach the foetal brain, where they react with foetal brain antigens. Implicated target antigens include Contactin Associated Protein 2 (CASPR2), the N-methyl-D-aspartate receptor (NMDAR) and various intracellular proteins. Potential therapeutic approaches include (1) therapeutic plasma exchange (TPE), (2) immunoadsorption (IA), (3) intravenous immunoglobulin therapy (IVIg), (4) blockade of the neonatal Fc receptor (FcRn), (5) peptidomimetics and (6) non-destructive monoclonal immunoglobulin G (IgG) with the same specificity as the pathogenic antibody. An overview of each approach can be found in Table A3.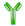 = Maternal anti-brain antibody; 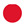 = Implicated antibody targets; 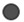 = Candidate treatment approaches.

The animal studies summarised in Table A2 have helped to strengthen the story of MAR autism. However, it is important to keep in mind the difficulty of comparing animal and human behaviours. It is possible that some or all of the maternal antibodies identified are mediators not specifically of autism but of neurodevelopmental disorder more generally. This issue is further complicated by changes in autism diagnosis over the past 30 years. Baird et al. (2006) [30] described a disparity between the recorded prevalence of childhood autism in South Thames, UK (38.9 per 10,000) and the prevalence measured using a narrower definition which combined clinical consensus with instrument criteria for past and current presentation (24.8 per 10,000). Research subject selection in many of the studies in Table A1, including Brimberg et al. (2013) [19], appear to have been based on the wider definition, meaning there might be inconsistencies depending on where and when the cohorts were recruited and when studied. This could be an explanation for some conflicting results discussed later in this article concerning CASPR2 antibodies (CASPR2-Abs). A simple schema of the placental transfer, the antigenic targets, and possible treatment approaches is shown in Figure 1.

## 3. Intracellular Antigen Targets

As mentioned previously a series of U.S. studies beginning in 2007 aimed to identify autoantibodies associated with MAR autism. The first of these (Zimmerman et al., 2007) [20] used the Western blotting procedure outlined in Table A1 to show that plasma IgG from mothers of autistic children display altered reactivity with rat brain proteins compared to controls. In subsequent studies Western blotting was used to identify protein bands that showed increased reactivity with plasma from mothers of children with autism. The pattern with the highest specificity for autism was paired reactivity to 37 and 73 kDa protein bands [21,22,23,24]. Combined results from Braunschweig et al.’s 2008 and 2012 studies (356 ASD mothers, 389 controls) revealed that paired 37/73 kDa reactivity was associated with a diagnosis of ASD in the child at an odds ratio of 39.4 and was absent in all control mothers [21,24]. Thus, this remains the most specific maternal biomarker of autism to date. In a mouse model Braunschweig et al. (2012) [25] demonstrated delayed growth, increased anxiety and shortened social interaction in offspring exposed in utero to IgG from mothers displaying paired 37/73 kDa reactivity. The following year, Bauman et al. performed the same experiment in rhesus macaques and observed compelling social abnormalities in the test group, accompanied by increases in brain size and white matter volume [31], which have been observed as features of both autism in general [32,33,34,35,36,37,38,39] and MAR autism specifically [40]. Despite this strong evidence for the association of autism with maternal antibodies to 37 and 73 kDa brain proteins, the pathological significance of these specific antibodies, which were transferred to the animals with polyclonal purified IgG, is still in question.

With a view to addressing this issue Braunschweig et al. (2013) [26] attempted to identify the antigens isolated in preceding studies. They used tandem mass spectrometry and peptide sequencing to determine the identity of the 37, 39, 73, 70 and 44 kDa proteins detected on Western blots. A range of different proteins were identified, including lactate dehydrogenase A (LDH-A), lactate dehydrogenase B (LDH-B), stress-induced-phosphoprotein 1 (STIP1), Y-box-binding protein 1 (YBX1), collapsin response mediator protein 1 (CRMP1) and cypin. These were confirmed as the targets for antibody binding by demonstration that pre-incubation of patient antibodies with the corresponding protein abrogated binding on the blots. All seven proteins have established functions in neurodevelopment, supporting their association with MAR autism. However, each is also involved in other processes, including glycolysis (LDH-A) [41] and gene transcription and translation (YBX1) [42]. Complete disruption of any these functions would be expected to have more dramatic and widespread effects than observed in MAR autism. Of course partial disruption of these functions may well give rise to a milder phenotype, and further study should consider this.

It is important also to mention that while a subset of antibodies can penetrate live cells to reach intracellular targets (perhaps particularly during neurodevelopment) [43], antibodies to intracellular antigens are relatively unlikely to be directly pathogenic, as their targets are inaccessible to circulating factors. This exposes a disadvantage of using foetal brain protein preparations rather than intact tissue or neurons to search for neuronal antibodies in the mothers. Moreover, a high proportion of all mothers (89% of mothers of children with autism and 70% of mothers of typically developing children) had antibodies to only one of the two proteins, reducing the likelihood that any one protein can be pathogenic in isolation. As the authors discuss, it is more likely that disease can be caused by autoantibody combinations [26]. Further work was required to address this issue and determine whether MAR antibodies can enter/be taken up by live neurons.

Further IgG transfer experiments conducted in 2016 and 2017 strengthened support for a pathogenic effect. Purified patient IgG containing 37/73 kDa antibodies was injected into the cerebral ventricles of mice in utero, and the brains were examined 2 h or 2 days later. In two papers [44,45] the authors showed that the IgG recognised radial glial cells, increased ventricular zone cell proliferation and subsequent cortical neurons and reduced dendritic spines. As in the previous studies from Braunschweig et al. [25] and Bauman et al. [31], this indicated potential pathogenicity of the IgG but did not prove that the 37/73 antibodies were responsible. To overcome this, Jones et al. (2018) [46] used an active immunisation approach against the antigens. The authors used the immuno-dominant B-cell epitopes of LDH-A, LDH-B, STIP1 and CRMP1 to produce an antigen-driven model based on the autism-specific 37/73 kDa band pattern. Differences observed in juvenile sociability and repetitive behaviours were interpreted as evidence that prenatal exposure to autoantibodies against these epitopes mediates behavioural changes in offspring. However, the enzyme-linked immunosorbent assays (ELISAs) done to confirm antibody generation produced very low antibody titres, ranging between 0.3 and 0.5 optical density (OD), and it was not clear whether the behavioural changes were due to the specific antibodies rather than, for instance, the result of formation of inflammatory immune complexes with the peptide antigens.

Thus, the role of antibodies to intracellular antigens in MAR autism requires further investigation. While they could have pathogenic significance, it is possible and perhaps more likely that they represent a more widespread, although relevant, immune response. Disease-specific patterns of autoantibody reactivity to intracellular antigens are epiphenomena in numerous autoimmune diseases and provide a valuable tool in clinical diagnosis [47]. Braunschweig et al. (2013) reported that maternal reactivity to any of the seven intracellular antigens was significantly associated with an outcome of autism in a cohort of 395 (OR = 3.26) and increased incidence of stereotypic behaviour was observed in children of mothers with LDH, CRMP1 and STIP1 reactivity, supporting the rhesus macaque experiments reported by Martin et al. (2008) [18]. These results suggest the potential of autoantibodies to these antigens to be biomarkers of autism but their pathogenic role needs further study.

## 4. Extracellular Antigen Targets

### 4.1. Contactin Associated Protein 2 (CASPR2)

Perhaps the most promising candidate pathogenic antigen to date is an antibody to Contactin Associated Protein 2 (CASPR2), a membrane protein complexed with the neuronal potassium channel (VGKC). CASPR2-Abs are not common in the general population (<1%) but are present in patients with an acquired form of neurological disease associated with VGKC loss of function and both peripheral and central neuronal hyperexcitability (Irani et al., 2010) [48]. Brimberg et al. (2016) [27] cloned antibodies directly from the B cells of mothers of autistic children and identified the target of one monoclonal antibody as CASPR2. This monoclonal antibody (C6) was isolated and injected into pregnant mice and produced offspring displaying autism-related histopathology and behaviours. Histopathologically, of particular note are the change in cortical thickness and decrease in cortical neuronal packing, as these are features that have been observed in postmortem studies of autism [49]. In a social interaction test C6-exposed mice spent equal time with an unfamiliar mouse, an unfamiliar object and empty space, while control antibody B1-exposed mice spent more time with the unfamiliar mouse. It is possible that these results represent a general lack of motivation to explore in C6-exposed mice, but this is perhaps less likely given that both groups of mice were equally interested in the unfamiliar object, which the authors argued increases the specificity of the behavioural changes for the social aspects of autism. C6-exposed mice also displayed stereotypic behaviour in the marble-burying test and decreased flexible learning in the clock maze test comparable to inflexible behaviour in autism. There were no confounding anxiety-like behaviours in the open field test. Overall, the histopathological changes, range of behavioural features and absence of the confounding anxiety-related behaviours observed in some other studies [25,28] make this one of the most convincing autism-like phenotypes produced in a MAR autism model to date. Brimberg et al. also reported plasma CASPR2 antibody reactivity in 37% of 53 ASD mothers whose serum was reactive with rodent brain tissue, compared to 8% of mothers of typically developing children and 12% of both non-brain-reactive ASD mothers and unselected women of child-bearing age. This supported CASPR2 as one possible antigenic target in MAR autism, but the positive rate in the controls was much higher than that currently found in neurological diagnostic laboratories (1–2%), raising some questions about assay specificity. Moreover, the brain-reactive sera only represented 10% of the larger cohort of ASD mothers [20]; these factors make it difficult to assess the significance of CASPR2 antibodies in ASD as a whole.

In a parallel study Coutinho et al. (2017) used assays in routine diagnostic use and tested large cohorts of gestational sera collected in the 1990s. CASPR2-Abs were not associated with autism (defined during the 1990s) but found in 4.4% of mothers of children with mental retardation and/or disorders of psychological development (MR/DPD) compared with 0.9% of all other pregnant mothers [50]. In the same year Coutinho et al. also described behavioural changes in mice exposed in utero to IgG from two patients with CASPR2-Ab neurological disorders [51]. IgG-exposed mice displayed decreased sociability in three-chamber and reciprocal social interaction tests and increased time spent in non-social activity (digging and grooming), with no confounding differences in motor coordination, locomotor activity or anxiety-like behaviours. Crucially, no differences were apparent in neonatal development or motor coordination, as assessed by the modified Fox battery, meaning changes in social behaviour could not be attributed to general deficits. Furthermore, the authors observed a 15–52% reduction in PSD-95 positive (glutamatergic) synaptic profile density in the prelimbic, infralimbic and somatosensory cortices of CASPR2-Ab-exposed mice, alongside a 16% increase in CD68/Iba+ cells (activated microglia). These findings might reflect a role for microglia in extensive pruning of aberrant glutamatergic synapses in CASPR2-Ab-exposed mice with disrupted CASPR2-mediated GluA1 trafficking. Both Coutinho et al. and Brimberg et al. describe a CASPR2-Ab-induced autism-like phenotype, but Coutinho et al. did not find CASPR2-Abs in patients with autism diagnosed in the 1990s; it is possible, therefore, that CASPR2-Abs are associated with the wider spectrum disorder rather than the classically defined disease.

Unrelated studies of embryonic lipopolysaccharide (LPS) [52] and 1-methyl-4-phenyl-1,2,3,6-tetrahydropyridine (MPTP) [53] exposure have demonstrated that once over-activated, microglia may remain in a state of dysregulation. Consistent dysregulation of microglial synaptic pruning and inflammatory microglial activation has been widely defined as a life-long feature of autism [54]. This is thought to contribute significantly to the unusually large size of autistic brains, a phenotype that is more dramatically observed in the MAR autism subgroup [40]. Thus, an impressive body of evidence, but not yet supported by an adequate case-control study, incriminates maternal CASPR2-Abs as one possible cause of autism.

Another aspect supporting the role of CASPR2 autoantibodies in MAR autism is the association of loss of function Contactin Associated Protein 2 gene (CNTNAP2) mutations with rare genetic forms of autism [55,56]. Table 1 presents parallels between models of CNTNAP2 deficiency disorder and maternal CASPR2 reactivity, which suggest a likely similarity between their presentations. Firstly, in CNTNAP2-mutated cells there is a reduction of neuronal surface CASPR2 function, which is thought to underlie the resulting disorder [57]. Fernandes et al. (2019) [58] showed that CASPR2 function is also reduced in neurons exposed to CASPR2-Abs. The authors incubated cortical neurons with IgG from patients with CASPR2 antibody encephalitis and observed a decrease in dendritic CASPR2 clusters, demonstrating a direct effect of autoantibodies on endogenous levels of neuronal surface CASPR2. Similarly, both Dawes et al. (2018) [59] and Giannoccaro et al. (2019) [60] showed that CASPR2-Abs changed CASPR2 expression and VGKC expression in mouse models. Secondly, both CNTNAP2 deficiency and CASPR2-Ab-related developmental disorder have presented histologically with abnormalities in neuronal migration. Penagarikano et al. (2011) [56] observed that CNTNAP2-knockout mice had high numbers of CUX1+ (layer II-IV cells) in cortical layers V and VI, a change that was seen almost identically in CASPR2-Ab-exposed mice (Coutinho et al., 2017) [51]. Notably, irregular lamination has also been evident in human autism brains postmortem [49]. Furthermore, Brimberg et al. (2016) [27] observed various cortical changes suggestive of aberrant neuronal migration in CASPR2-Ab-exposed mouse foetuses (Table 1). Third, Anderson et al. (2012) [61] showed that RNAi knockdown of CASPR2 in pyramidal neurons gave rise to decreased dendritic spine arborisation, which was mirrored in Brimberg et al.’s gestational transfer model when CASPR2-Ab-exposed pyramidal neurons displayed reduced dendritic tree complexity [27]. Finally, both CASPR2-deficient [56] and CASPR2-Ab-exposed [27] mice exhibited a reduction in GABAergic parvalbumin positive (PV+) hippocampal neurons.

### 4.2. N-methyl-D-aspartate Receptor (NMDAR)

There has been a smaller body of relevant evidence looking at the role of antibodies against the NMDA receptor (NMDAR-Abs) in the development of neuropsychiatric disorder, including autism. While the links to autism are not currently strong or specific, recent work by Jurek et al. (2019) has prompted further consideration of this potential target.

The first evidence on NMDAR antibodies in neurodevelopmental disorder came in 2009 from Lee et al. The authors mated mice after immunisation with the DWEYS pentapeptide, which is present within the NR2A and NR2B subunits of the NMDAR. Antibody-exposed embryological day (E) 15 foetuses displayed histological brain abnormalities, including cortical thinning, which persisted into adulthood in mice exposed to high antibody titres. Pups exposed to antibody in utero exhibited a dose-dependent delay in emergence of the negative geotaxis reflex, and adult mice exposed to high but not low levels of NMDAR-Abs performed worse in fear extinction, novel object recognition and topological tasks [62]. These data suggested a causative link between maternal NMDAR-Abs and persisting developmental disorder in the child. The correlation between antibody titre and extent of neurological deficit increases our confidence that this effect is antibody mediated. However, these studies were designed to address a subgroup of patients with systemic lupus erythematosus (SLE) in whom the same group had already reported antibodies against NR2. These antibodies could contribute to neuropsychiatric disorders in the mothers with SLE and to the high incidence of learning disorders in their children, but may not be relevant to the wider population; Coutinho et al. (2017) looked at NMDAR-Abs using a combination of NR1 and NR2 subunits and did not find these antibodies to be more frequent in mothers of autistic children than in the age- and parity-matched controls [50].

Nevertheless, Jurek et al. (2019) [29] have reported the effects of maternal antibodies to the NR1 subunit of the NMDAR, which they found to be present in around 1% of apparently healthy pregnant women. NMDAR-Abs are associated with a well-recognised form of autoimmune encephalitis associated with psychiatric disturbance, seizures, cognitive disturbance and movement disorders, most commonly in children and younger adult females. In these patients the antibodies cause loss of surface NMDARs by internalisation resulting in NMDAR hypofunction, although it is not clear how this results in the complex clinical features of the disease [63,64,65]. Only a few offspring of mothers with this disease have been reported and interpretation of their defects is further complicated by the very substantial pharmacological and immunological treatments the mothers would have been exposed to during their pregnancies [66,67,68,69,70,71]. However, it is likely that NMDAR-Abs at a level that did not cause symptoms in the mother could affect development. Jurek et al. established a mouse model in which recombinant human monoclonal NR1 antibodies, cloned from two patients with acute NMDAR-Ab encephalitis, were injected mid-gestationally at days E13 and E17 into the dams, with controls injected with isotype-matched control IgG. At birth human IgG antibodies were detected bound to the neuropil of the offspring’s hippocampi, and the offspring had reduced synaptic NMDAR densities, lower bodyweights, increased mortality and delayed neurodevelopment, which were measured via the righting reflex, cliff avoidance reflex and negative geotaxis reflex. There was also impaired pre-pulse inhibition and reduced anxiety, persisting into adulthood. Observations of decreased bodyweight [25] and impaired neurodevelopmental reflexes [16,25] were similar to those seen in gestational transfer models of autism, and motor delay is common in humans with the disorder. However, the findings in this study could relate to a spectrum of behavioural disorders, not necessarily autism.

With a view to clinical translation of their research, the authors compared serum NR1 reactivity in mothers of children with psychiatric disorder and mothers of typically developing children. They found slightly higher titres of NR1-reactive IgG in the former group, with no specific disorder being favoured. These data warrant further investigation involving measurement of mid-gestational and neonatal antibody titres and long-term follow-up in a large cohort. However, Coutinho et al. (2017) did perform such a study and found low levels of NMDAR-Abs to be present in both mothers of children with neurodevelopmental disorders (8% in MR/DPD and 2% in ASD) and age- and parity-matched controls (3–5%), with no significant difference between them [50]. It is still possible that NMDAR-Abs at low levels can affect development in utero, but that other factors are required to increase susceptibility to the antibody-mediated changes. Overall, pathogenic NR1 as well as CASPR2 antibodies may account for a proportion of MAR autism cases, but there is much more information required.

## 5. Why Do Mothers Develop Brain Reactive Autoantibodies?

There are a number of potential explanations for the presence of brain reactive autoantibodies in maternal serum. Autism is well-established to be associated with a family history of autoimmune disease, as well as certain MHC haplotypes and gene polymorphisms [6,7,8]. Thus, one contributing factor is almost certainly a general breakdown of maternal immune tolerance. In line with this, Heuer et al. (2011) found a strong association between paired 37/73 kDa maternal reactivity and presence of the MET C allele, which correlated with reduced expression of the immune-regulatory MET receptor tyrosine kinase. The authors also found decreased levels of the regulatory cytokine IL-10 to be associated with presence of MET C and reduced MET protein expression [72]. These results indicate that genetics play a part in the breakdown of maternal immune tolerance in MAR autism. Environmental factors might also play a role, for example cross-reactivity with infectious agents can initiate autoimmunity [73].

Another interesting possibility is that autoantibodies in MAR autism are driven by their antigen, which is presented on the surface of foetal cells in the maternal circulation. In the study of AMC Saxena et al. (2017) [74] characterised an anti-foetal-AChR monoclonal isolated from an MG patient and found a high replacement to silent (R/S) mutation ratio among the genes encoding the variable regions, indicating antigen-driven somatic hypermutation. Characterisation of autoantibodies in MAR autism could elucidate whether a similar mechanism might be at play, and longitudinal study comparing plasma reactivity of mothers before and during pregnancy could be informative. Of course, reaction to circulating foetal cells might be more likely on a background of impaired immune tolerance and could be more likely with increasing parity and age.

More complicated is the issue of antibodies to intracellular antigens. As discussed previously, these might be disease-specific epiphenomena in MAR autism, just as they appear to be in SLE and a number of other autoimmune diseases. In SLE, the autoantibodies are almost always somatically hypermutated, indicating that they are produced in contact with antigen; this appears to be the result of presentation of intracellular proteins by apoptotic bodies due to defective clearance of apoptotic cells [47]. It is thinkable that a similar mechanism is at play in autism, involving exposure of the mother to foetal apoptotic bodies expressing intracellular antigens. Although speculative, it is possible that excess apoptotic bodies are produced in the foetal brain in MAR autism, owing to damage caused by the pathogenic neuronal surface antibodies, the inflammatory state associated with autism, a defect in an apoptosis clearance pathway (often seen in autoimmune disease), or a combination of factors. Furthermore, studies by Bischoff et al. (2004) [75] and van Wijk et al. (2000) [76] used transmission electron microscopy (TEM) and quantitative polymerase chain reaction (qPCR) to reveal foetal apoptotic bodies in maternal plasma, providing evidence that apoptotic bodies cross the placenta to reach maternal circulation. This might be an interesting avenue to explore as a parallel between MAR autism and other autoimmune diseases, such as SLE, in which similar disease-specific patterns of autoantibodies to intracellular antigens exist.

## 6. Identifying Autism-Specific Autoantibody Patterns as Biomarkers of Maternal-Autoantibody-Related Autism

The evidence available to date has begun to build a picture of the antibody profiles that might be associated with MAR autism. However, if there is to be clinically valuable progress in the direction of screening for high risk pregnancies and prophylactic treatment, far more specific biomarkers need to be identified.

Braunschweig et al. (2013) [26] sought to address this, reporting that certain combinations of their seven proteins were found only in plasma from mothers of autistic children. An LDH/STIP1/CRIMP1 combination had the highest specificity (5% of ASD mothers as against no control mothers). This pattern corresponds to the highly autism-specific 37/73 kDa band pattern previously described. However, specificity of some combinations might be attributable to the low numbers of patients studied. Larger-cohort studies are needed and all biomarker studies of this type need independent replication in well-defined cohorts and, importantly, in gestational rather than postpartum samples.

Edmiston et al. (2018) [77] considered epitope specificity of the same seven antigens in MAR autism (*n* = 55) and control (*n* = 30) groups. Overlapping peptide microarrays were incubated with pooled plasma from samples displaying reactivity to at least one of the full-length proteins (*n* = 29). 75 candidate peptides were then used to screen the entire cohort and maternal reactivity was observed exclusively in mothers of autistic children for certain peptides, including DCIIIVVSNPVDILT (LDH-B), which demonstrated high specificity for autism (9.1% MAU vs. 0% MTD). Interestingly, some epitopes, including DCIIIVVSNPVDILT, only reacted with plasma from the mothers of children with severe autism. If verified in a larger cohort, a grading system of biomarkers could prove a useful prognostic tool. Mechanistically, this severity-dependent reactivity might reflect epitope spreading in anti-intracellular antibodies, a process catalysed by continuous autoimmune tissue destruction [78]. Findings that only a small amino-acid change can differentiate a peptide that reacts in severe cases from one that reacts in mild cases support this hypothesis [77].

Some of these biomarkers might be useful in graded assessment of patient-specific risk following plasma identification of CASPR2 or another pathogenic antibody.

## 7. Multiple Hit Model

Autism is a highly complex disorder with multiple risk factors other than autoreactivity. A multiple hit model takes into account potential convergence of diverse risk factors to produce a predicted outcome of ASD. Some of the autoantibody biomarker candidates described show a trend towards very high specificity for autism and severe cases in particular [26,77]. However, for patients presenting with less specific autoantibody biomarker patterns, inclusion of further genetic and/or environmental criteria in assessment of the risk-adjusted benefit of treatment might help identify further high-risk patients. Crucially, in 2019 a genome-wide association study (GWAS) recruited a significantly larger cohort than previous such studies (18,381 autism-affected individuals and 27,969 controls) and identified five significant loci as the first common individual risk variants for the disorder [79]. This study looked at the ASD population in general but future work might consider (via GWAS) whether children affected by MAR autism are more likely to have autism risk genes than TD children of women with anti-brain reactivity. Furthermore, in light of the earlier discussion the MET C allele will likely serve as a helpful biomarker of MAR autism. The vision is to incorporate genetic biomarkers into MAR autism biomarker patterns, enabling more mothers to receive accurate predictions of their child’s outcome.

A similar approach could be taken to maternal immune activation (MIA) during gestation. Association studies might consider any correlations between outcomes of MAR autism and maternal infection during pregnancy or interleukin 17A (IL17A) in gestational plasma samples. In poly(I:C)-evoked MIA, IL17A is increased in maternal blood and foetal brain and studies suggest its action on microglial interleukin 17 receptor A (IL17RA) to provoke an inflammatory response [80]. Synergy between IL17-mediated and CASPR2-mediated microglial activation might make these two a very harmful combination. Furthermore, IL17A levels are also raised in certain MAR autism-associated autoimmune diseases including rheumatoid arthritis and psoriasis.

The single best predictor of MAR disorder seems to be occurrence of disorder in a previous pregnancy [11,12,16]. Thus, mothers with reactive serology should immediately be placed into a higher risk group if they have an existing child with ASD or a related neuropsychiatric disorder. Nevertheless, we want to emphasise that while there may be a substantial number of cases with a MAR component, typical autism and the wider ASD spectrum are likely to have different causes with individual or combinations of genetic, environmental and immunological mechanisms.

## 8. Approaching Treatment of Maternal-Autoantibody-Related Autism

Should appropriate gestational biomarkers be defined, we might eventually begin conversation on prophylactic therapy for MAR autism. Therefore, we briefly review some potential treatment options (see also Figure 1). The “gold standard” treatment of most autoimmune conditions remains immunotherapies that reduce antibody levels or, in some cases, target effector mechanisms. However, global immunosuppression poses a serious risk to a pregnant woman and could both jeopardise her health and risk neurodevelopmental disorder in her child [5]. Table A3 considers six treatments that have had success in the context of autoimmune diseases, either clinically or in preclinical testing, and may be of use in MAR autism.

The most complete and fastest way of reducing serum antibody levels is to use therapeutic plasma exchange (TPE), which replaces patient plasma with a substitute protein mix. John Newsom Davis et al. used plasma exchange followed two weeks later with intravenous immunoglobulin injection (IVIg) to prevent AMC in the child of a pregnant MG patient with a history of two affected pregnancies (unpublished data; the woman was case 6 in Riemersma et al., 1997 [13]); this was the first successful attempt at prophylaxis for a MAR disorder.

Of course, immediacy of solution is important in a life-threatening disorder like AMC, but for MAR autism, an autoantibody-specific treatment to maximise risk-adjusted benefit might be pursued. Options include antigen-specific immunoadsorption (IA), which has had preclinical success in MG [81], and peptidomimetics, which have various limitations but are constantly developing [82,83].

Finally, there are two further experimental approaches detailed in Table A3 that are worth mentioning. The first is blockade of the neonatal Fc receptor (FcRn), which has been shown to reproduce specific mechanisms of IVIg at lower doses with fewer adverse events [84]. FcRn blockade is currently in human-phase trials for MG, immune thrombocytopenia, subcutaneous formulation, pemphigus vulgaris and chronic inflammatory demyelinating polyneuropathy [85,86,87]; it also has potential for preventing placental transfer. The second approach is use of non-destructive monoclonal IgG. Here, antibodies with the same specificity as the pathogenic antibody are manufactured to lack effector functions, while retaining FcRn-binding properties and thus placental transfer; these antibodies compete with the pathogenic antibody for foetal antigen, but are not themselves pathogenic. This approach is highly specific, and has been confirmed as efficacious in humans for treatment of foetal/neonatal alloimmune thrombocytopaenia (FNAIT) [88,89].

## 9. Defining the Therapeutic Window

In any attempt at prophylaxis, it would be important to define a tight therapeutic window to minimise exposure of mother and foetus to treatment risks. There are three aspects to consider: When the IgG antibodies are transferred to the foetus and when the developing foetus is most at risk, which is partly dependent on the timing of blood–brain barrier (BBB) closure. First, the start of placental IgG transfer is thought to be approximately 13 weeks, with IgG reaching maternal levels in the baby by birth [90]. Thus it would be important to reduce maternal levels before 13 weeks and over the following months. The duration of treatment, would depend on when the developing foetus was most susceptible. Second, regarding the most vulnerable time for the developing brain, there are data in mice but very little in humans. Mouse studies involving administration of infrared-labelled antibody to pregnant mice have demonstrated dramatic drops in antibody penetration at E16.5–E17.5 (Braniste et al.) [91] and E15.5–E16.5 (Daneman et al.) [92], suggesting BBB-tightening can be pinpointed to a particular day. Studies by Hallman et al. (1995) [93] and Daneman et al. (2009) [94] found the loss of plasmalemma vesicle associated protein (PLVAP) coincided with the tightening of the BBB at E17.5 in mice. Antibody against human PLVAP has been validated for Western blot and could be used to measure disappearance of PLVAP in an in vitro model of the BBB. This is an area that warrants further work, specifically translation from animal to human studies since human development is very different from mouse. Third, the timing would depend partly on the nature of the antibody target and when it would be most likely to be detrimental. There are many unknowns.

As mentioned above, Newsom-Davis et al. prevented AMC with a treatment cycle that began with TPE at 12 weeks (unpublished data). In maternal lupus, Lee et al. (2009) [62] reported a dose-dependent effect of maternal anti-NMDA/anti-dsDNA antibodies on foetal behaviour, suggesting that later initiation of treatment might be acceptable, depending on the uncharacterised relationship between antibody dose and neurodevelopmental impairment in MAR autism. However, avoiding any risk of pathogenic brain reactivity should remain the primary concern.

## 10. Conclusions

In summary, a prophylactic treatment approach to MAR autism administered within a tightly defined gestational period, is an attractive option, but remains futuristic until the pathogenic autoantibodies and disease biomarkers are adequately characterised. CASPR2 is arguably the most promising candidate autoantibody target identified so far. Further, a mechanism related to apoptotic bodies provides a possible explanation for the unexplained association of MAR autism with autoantibodies targeting specific intracellular neuronal antigens, with these antibodies showing potential as the basis of graded, disease-specific biomarkers even if proved not to be pathogenic.

Overall, feasibility of treatment is conceivable in a subset of mothers testing positive for pathogenic autoantibodies, alongside a convincing pattern of autism-specific biomarkers. A case-by-case assessment of the risk-adjusted benefit of treatment could look at autoantibody-related, genetic and inflammatory biomarkers, among others. However, autism is a complex disorder with hundreds of risk factors, making it impossible to design criteria that will predict all outcomes of autism-associated serology with accuracy. It may be that MAR autism biomarkers will be of use primarily in informing advice on early behavioural intervention strategies that can reduce core autism symptoms [95]. In these cases, accurate diagnosis could be aided by foetal MRI to detect increased brain size and other brain changes associated with MAR autism.

## Figures and Tables

**Table 1:** Parallels between CNTNAP2 deficiency and anti-fetal CASPR2 reactivity.

CNTNAP2 Deficiency	CASPR2-Autoantibody-Related Developmental Disorder
CNTNAP2 mutation causes reduction of CASPR2 function [57]	Fernandes et al. (2019) [58]: Incubation of cortical neurons with IgG from patients with CASPR2-Ab encephalitis caused a decrease in dendritic CASPR2 clusters. First demonstration of a direct effect of autoantibodies on endogenous levels of neuronal-surface CASPR2. Dawes et al. (2018) [59] and Giannoccaro et al. (2019) [60]: CASPR2-Abs changed CASPR2 expression and VGKC expression in mouse models.
Penagarikano et al. (2011) [56]: Cntnap2-knockout mice displayed abnormalities in neuronal migration, indicated by high numbers of CUX1+ (layer II-IV cells) in cortical layers V and VI.	Coutinho et al. (2017) [51]: CASPR2-Ab-exposed adult mice had more CUX1+ cells in layers V and VI. Brimberg et al. (2016) [27]: CASPR2-Ab-exposed adult mice displayed cortical thinning, reduced numbers of proliferating cells in the developing cortex and reduced distances between neurons, likely reflecting aberrant migration of developing neurons.
Anderson et al. (2012) [61]: RNAi knockdown of CASPR2 in pyramidal neurons gave rise to decreased dendritic spine arborisation.	Brimberg et al. (2016) [27]: C6 (CASPR2-Ab) exposure in utero gave rise to a decrease in dendritic tree complexity in hippocampal pyramidal neurons.
Penagarikano et al. (2011) [56]: CASPR2-deficient mice displayed a 20% reduction in hippocampal GABAergic PV+ neurons.	Brimberg et al. (2016) [27]: C6 exposed mice had fewer GABAergic PV+ neurons in the hippocampus.

Abbreviations: Cntnap2 = Contactin associated protein 2 gene. CASPR2 = Contactin associated protein 2. CASPR2-Ab = CASPR2 antibody. VGKC = Voltage-gated potassium channel. CUX1 = Cut Like Homeobox 1. PV+ = Parvalbumin positive.

## Appendix A

**Table A1:** Summary of studies associating maternal antibodies with neurodevelopmental disorder in the child.

Author	Findings	Antibody Target	Cohort
Warren et al. (1990) [10]	6 of 11 mothers with an autistic child had antibodies reactive to lymphocytes of the child.	Human lymphocytes	MAU = 11 All mothers had an autistic child 6 years of age or younger. Plasma samples taken after recruitment
Since lymphocyte antigens can be shared with neuronal antigens, the study indicated that maternal antibodies might be associated with the development of autism.			
Barnes et al. (1995) [11]	Four children with AMC born to an initially asymptomatic mother and separate unrelated fathers The mother developed muscle weakness following her first pregnancy and was diagnosed with MG after her fourth.	Unclear at this stage	1 mother with 4 pregnancies affected by AMC and a subsequent diagnosis of MG
It was striking that the diagnosis of AMC was made only after the fourth affected child. A very high risk of recurrence in maternal MG-associated AMC is evident (also in Vincent et al., 1995).			
Vincent et al. (1995) [12] and Riemersma et al. (1997) [13]	Serum from a mother of children affected by a neuromuscular disorder contained a high titre of antibodies that selectively inhibited the foetal form of acetylcholine receptor.	Acetylcholine receptors (antibodies inhibiting function of foetal isoform)	1 healthy mother with 6 consecutive pregnancies affected by AMC Serum was taken before the 6^th^ pregnancy.
First study to implicate clearly maternal antibodies as a cause of developmental disorder in the children of unaffected mothers Implications for a pathogenic role of the maternal antibodies: 1. Recurrence of AMC in 6 pregnancies makes a genetic cause unlikely. 2. Foetal movements were observed in the pregnancies until 15–18 weeks, the time at which foetal IgG levels begin to rise rapidly. 3. Repeated plasma exchange from 10 weeks gestation prolonged foetal movements.			
Zimmerman et al. (2007) [20]	Antibodies in the serum of mothers of autistic children recognised different foetal but not adult rat brain proteins compared to controls.	Adult and foetal rat brain protein preparations	MAU = 11 MTD = 10 Plasma samples taken after recruitment
Western blotting procedure as in numerous following studies: 1. Brain protein preparation was gel electrophoresed, then transferred onto nitrocellulose membrane. 2. Nitrocellulose membranes were incubated in maternal plasma, then incubated with secondary antibody before imaging. Crude membrane proteins are not ideal as the they expose both intracellular and extracellular sequences, and also gel electrophoresis requires denaturation of the proteins.			
Braunschweig et al. (2008) [21]	Increased plasma reactivity to 37 kDa band in mothers of autistic children Paired reactivity to 37 and 73 kDa bands found exclusively in mothers of autistic children	Foetal human brain protein preparation	MAU = 61 Controls: MTD = 62 MDD = 40 Plasma samples taken after recruitment
There is inconsistency in the bands identified by the five immunoblotting studies. However, when results from Braunschweig’s 2008 and 2012 studies are combined, the 37/73 kDa band pattern achieves a highly specific association with ASD, yielding an odds ratio of 39.4.			
Croen et al. (2008) [22]	Increased plasma reactivity to a 39 kDa band in mothers of autistic children Paired reactivity to 37 and 73 kDa bands found exclusively in mothers of children with early onset autism	Foetal human brain protein preparation	MAU = 84 Controls: MTD = 160 MDD = 49 Plasma samples taken after recruitment
Singer et al. (2008) [23]	Increased plasma reactivity to 36, 39, 61 and 73 kDa bands in mothers of autistic children	Human and rat foetal brain protein preparation	MAU = 100 MTD = 100 Plasma samples taken after recruitment
Braunschweig et al. (2012) [24]	Increased plasma reactivity to 37, 39 and 73 kDa bands in mothers of autistic children 37/73 kDa paired reactivity strongly increased in mothers of autistic children	Foetal monkey brain protein preparation	MAU = 272 Controls: MTD = 185 MDD = 102 Plasma samples taken after recruitment
Braunschweig et al. (2013) [26]	Tandem mass spectrometry and peptide sequencing were used to identify antigen candidates for 37, 39, 73, 70 and 44 kDa proteins. LDH, YBX1, cypin, STIP1, CRMP1 and CRMP2 were confirmed as autoantibody targets in binding inhibition Western blot studies.	Foetal monkey brain protein preparation	MAU = 246 MTD = 149 Plasma samples taken after recruitment
Brimberg et al. (2013) [19]	Plasmas from mothers of autistic children were four times more likely to contain anti-brain antibodies than control plasmas (10.5% vs. 2.6%).	Mouse brain sections	MAU = 2431 MTD = 653 Plasma samples were from blood banks and not mid-gestational.
First large cohort study and thus important evidence that mothers of an autistic child have an elevated frequency of anti-brain antibodies Most reliable indicator of the incidence of MAR autism			
Brimberg et al. (2016) [27]	Plasma reactivity to CASPR2 was found in 37% of ASD mothers displaying brain-reactive serology compared to 12% of ASD mothers lacking brain-reactive serology.	Mouse brain in vivo Caspr2-transfected HEK-293T cells	MAU with reactive serology = 53 MAU lacking reactive serology = 63 Plasma samples as above
Coutinho et al. (April 2017) [50]	CASPR2-Abs found more frequently in mothers of children with MR/DPD (7/171; 4%) than control mothers (1/171; 1%) No excess of CASPR2-Abs in mothers of children with autism (1/95; 1.1%) compared to control mothers (2/95; 2.1%). No excess of NMDAR antibodies compared to controls in mothers of children with neurodevelopmental disorders (10/171; 4% vs. 9/171; 5%) or autism (2/95; 2% vs. 3/95; 3%).	Live human embryonic kidney cells transfected with cDNA encoding CASPR2 or NMDAR	Gestational week 14–18 mothers of children with mild or unspecified neurodevelopmental disorders (n = 171, cases) and mothers whose child had not received this diagnosis (n = 171, controls) Gestational week 14–18 mothers of children with ASD (n = 95, cases) and mothers whose child had not received this diagnosis (n = 95, controls) Serum samples from the pregnancy screening biobank at the Danish State Serum Institute
Jurek et al. (2019) [29]	Mothers of children with psychiatric disorders had slightly higher titres of NR1-reactive IgG compared to mothers of unaffected children.	Live HEK cells over-expressing native human NR1 protein	120 healthy mothers of children with psychiatric disorders; 105 healthy control mothers of unaffected children Plasma samples taken 4–20 years after the pregnancy
No association with any specific psychiatric disorder			

Abbreviations: MAU = mothers of a child with autism spectrum disorder. MTD = mothers with only typically developing children. MDD = mothers of a child with developmental delay. AMC = arthrogryposis multiplex congenita. MG = myasthenia gravis. ASD = autism spectrum disorder. LDH = lactate dehydrogenase, YBX1 = Y-box-binding protein, STIP1 = stress-induced phosphoprotein 1. CRMP1, CRMP2 = collapsin response mediator proteins 1 and 2. MAR = maternal autoantibody related. CASPR2 = contactin associated protein 2. HEK = human embryonic kidney. MR/DPD = mental retardation and/or disorders of psychological development. CASPR2-Abs = CASPR2 antibodies. NMDAR = *N*-methyl-D-aspartate receptor.

**Table A2:** Summary of animal studies on maternal antibodies causing neurodevelopmental disorder.

Author	Findings	Cohort
Jacobson et al. (1999) [15]	Plasma from four AChR antibody-positive women injected into pregnant mouse dams: foetuses displayed deformities resembling AMC.	4 AChR antibody-positive women with histories of severe AMC in their babies Control plasma obtained from healthy laboratory workers (including 3 women with recent pregnancies) and therapeutic plasma exchange (other neurological diseases control)
First to establish passive transfer animal model for study of maternal antibody-mediated developmental disorder		
Dalton et al. (2003) [16]	Plasma from a mother of an autistic child and a child with a severe specific language disorder injected into pregnant mouse dams: offspring displayed a slowed righting reflex, decreased exploration and decreased performance on the multiple static rods test.	1 mother of 3 children: 1 TD, 1 with autism and 1 with a severe specific language disorder; 4 mothers with between 1 and 4 typically developing children; Plasma samples taken after recruitment
First causative study indicating that maternal factor can induce behavioural disorder in offspring		
Martin et al. (2008) [18]	Prenatal exposure to purified pooled IgG from mothers of autistic children produced stereotypies and hyperactivity in rhesus macaques.	MAU = 21 All mothers had at least two children with autism; MTD = 7 All mothers had at least two typically developing children; Plasma samples taken after recruitment
No changes in sociability were observed. However, the changes observed are particularly compelling in a primate model.		
Lee et al. (2009) [62]	NMDAR (NR2) immunisation, with NMDAR antibodies present before and throughout gestation NMDAR antibody-exposed E15 foetuses displayed histological brain abnormalities, including cortical thinning, which persisted into adulthood in offspring of dams with high titres. These pups were slow to develop the negative geotaxis reflex. When adult offspring were impaired in fear extinction, novel object recognition and topological tasks.	
Antibodies in this model cross-reacted with NR2A and NR2B. However, mouse NR2A expression is thought to be strictly postnatal so the observed phenotype was most likely due to antibodies targeting NR2B. In humans, NR2A expression has been detected in the foetal neocortex. Supports a role for NMDAR antibodies in disruption of foetal cortical development, but relationship to autism not clear		
Singer et al. (2009) [28]	Prenatal exposure to purified pooled IgG from mothers of autistic children produced hyperactivity, sociability alterations and anxiety-like behaviour in mice.	MAU = 63 All children judged to have moderate to severe adaptive or cognitive deficits by formal testing TD = 63 Plasma samples taken after recruitment
Braunschweig et al. (2012) [25]	Purified IgG from mothers of autistic children with paired 37/73 kDa band reactivity was injected into pregnant mice: Offspring displayed anxiety-like behaviour and delayed physical and sensory-motor development.	MAU = 3 (selected for paired 37/73 kDa reactivity) MTD = 3 Plasma samples taken after recruitment
No significant changes in sociability		
Bauman et al. (2013) [31]	Purified IgG from mothers of autistic children with paired 37/73 kDa band reactivity was injected into pregnant rhesus macaques: Offspring were treated with heightened protectiveness by their mothers, and on maturing they displayed increased approach of familiar peers (was not reciprocated and didn’t lead to sustained social interactions) and inappropriate approach of unfamiliar peers. Male offspring also had enlarged brains and increased white matter volume most pronounced in the frontal lobes.	MAU = 4 mothers (selected for paired 37/73 kDa reactivity) MTD = 5 mothers (selected to lack 37 and 73 kDa reactivity) Plasma samples taken after recruitment Plasma samples taken after recruitment
These were very compelling data and the first demonstration of aberrant social development in non-human primates exposed to autism-associated anti-brain antibodies. Increased brain size and white matter volume have been consistently reported in autism [32,33,34,35,36,37,38,39] and have been specifically reported in the MAR autism subtype [40]. The study was limited by its small sample size (8 macaques received MAU IgG, 8 received control IgG).		
Brimberg et al. (2016) [27]	Monoclonal C6 (CASPR2 antibody) derived from mother of autistic child injected into pregnant mice: offspring displayed differences in sociability, repetitive behaviour and flexible learning.	Monoclonal CASPR2 derived from 1 mother of an autistic child Control = Non-brain-reactive human antibody, B1
Martínez-Cerdeño et al. (2016) [44]	Purified IgG from mothers of autistic children with paired 37/73 kDa band reactivity was injected into pregnant mice: IgG recognised radial glial cells, increased subventricular zone and extra-cortical cell proliferation and increased both the size and weight of adult brains and the size of adult cortical neurons.	MAU = 4 (selected for paired 37/73 kDa reactivity) MTD = 5 (selected to lack 37 and 73 kDa reactivity) Plasma samples were from the CHARGE study at the University of California and were taken 1–5 years after pregnancy
Ariza et al. (2017) [45]	Purified IgG from mothers of autistic children with paired 37/73 kDa band reactivity was injected into pregnant mice: IgG reduced dendritic spine number and density in the frontal and occipital cortices.	MAU = 1 (selected for paired high-titre 37/73 kDa reactivity) MTD = 1 (selected to lack 37 and 73 kDa reactivity) Plasma samples were from the CHARGE study at the University of California and were taken 1–5 years after pregnancy
Coutinho et al. (October 2017) [51]	IgG from two patients with CASPR2-antibody encephalitis injected into pregnant mice: Adult offspring showed social interaction deficits, abnormally located glutamatergic neurons in layers V-VI of the somatosensory cortex, microglial overactivation and a reduction in glutamatergic synaptic profile density in the prefrontal and somatosensory cortices.	2 male patients with CASPR2-antibody encephalitis 3 healthy age- and sex- matched individuals
Jones et al. (2018) [46]	Antigen-driven model of MAR autism based on the ASD-specific 37/73 kDa band pattern: offspring of immunised dams showed differences in juvenile sociability and repetitive behaviours.	
The ELISAs done to confirm antibody generation produced low antibody titres, ranging between 0.3 and 0.5 OD; No attempt was made to verify that the effect was antibody-mediated rather than the result of formation of inflammatory immune complexes with the peptide antigens. Mixed results in behavioural tests		
Jurek et al. (2019) [29]	Cloned human antibodies were injected into pregnant mice: Offspring had reduced synaptic NMDAR densities, increased mortality, altered physiological functions during early development (elevated blood pH, reduced weight gain) and impaired neurodevelopment. Behavioural changes persisted into adulthood.	IgG1 cloned from 2 female patients with acute NMDAR encephalitis (obtained during periods of disease exacerbation) Control = non-brain-reactive human IgG1
Findings could relate to a spectrum of behavioural disorders, not necessarily autism.		

Abbreviations: MAU = mothers of a child with autism spectrum disorder. MTD = mothers with only typically developing children. MDD = mothers of a child with developmental delay. NMDAR = N-methyl-D-aspartate receptor. CASPR2 = contactin associated protein 2. ASD = autism spectrum disorder. ELISA = enzyme-linked immunosorbent assay. OD = optical density.

## Appendix B

**Table A3:** Potential therapeutic avenues.

Treatment	Past Success	Potential in MAR ASD
Commonly used immunotherapeutics, e.g., steroids, azathioprine, mycophenolate, cyclosporine, cyclophosphamide	Commonly used to treat multiple autoimmune disorders, including MG and GBS Azathioprine is frequently used as a “steroid sparing” agent and can be safely used in pregnancy, but its action is slow (Palace with Newsom-Davis et al., 1998) [96].	Only azathioprine is considered safe enough and its slow speed of action would mean the need to start treatment well before any pregnancy. Other drugs in common use are not considered safe in pregnancy or the information is insufficient to assess risks.
Therapeutic plasma exchange (TPE)	Commonly used to treat multiple autoimmune disorders including MG and GBS. Was used successfully in combination with IVIg to prevent AMC (John Newsom-Davis, unpublished results)	Relatively safe, effective and well tested procedure for treatment of autoimmune disease. Depletion of maternal antibodies might increase risk of maternal infection and interfere with materno–foetal IgG transfer, both of which are risk factors for neurodevelopmental disorder [97].
Immunoadsorption (IA)	Increasingly recognised as an alternative to TPE and has been used to treat various neurological disorders [98] Success in MG: (1) Schneider-Gold et al. (2016) [99]; (2) Lagoumintzis et al. (2014) [81]	Removes only antibodies, meaning other plasma components are not depleted Risk of adverse events is lower than in TPE. This is an important consideration in pregnancy. Semi-selective—Increased selectivity of adsorbents could reduce the removal of protective antibodies, but no evidence yet supports this concern.
Intravenous immunoglobulin (IVIg)	Established as a first-line or adjunctive treatment for treatment of GBS, multifocal motor neuropathy and chronic inflammatory demyelinating polyneuropathy Second-line for dermatonyostitis, MG, and stiff-person syndrome [100] Was used successfully in combination with TPE to prevent AMC (John Newsom-Davis, unpublished results)	No depletion of protective antibodies Non-invasive Doesn’t completely remove pathogenic autoantibodies (IVIg may not be sufficient alone in MAR autism) Significant risk of adverse events [101]
FcRn blockade	FcRn blockade reduces serum levels of antibodies by inhibiting the FcR-dependent recycling of IgG back into the serum, thus allowing its degradation [102,103]. ARGX-113 (efgartigimod): Uses argenx proprietary ABDEGTM technology; currently in human-phase trials for MG, primary immune thrombocytopenia, pemphigus vulgaris and chronic inflammatory demyelinating polyneuropathy [85]. HL161/IMVT-1401 (formerly RVT-1401): a humanised anti-FcRn monoclonal Ab in development by HanAll BioPharma/Immunovant; currently in human-phase trials for Grave’s ophthalmology, MG, warm autoimmune haemolytic anaemia and neuromyelitis optica [86,87].	Reproduction of specific mechanisms of IVIg at lower doses (25–50× lower for Abdegs) [84] The worldwide shortage of IVIg increases the demand for alternative options [104]. Fewer adverse events than IVIg due to the lower infusion volume and more targeted approach [84] No risk of blood-product-related infection. No modulation of B cells, plasma cells or T cells, as occurs with IVIg. Specific blockade of the placental FcRn would preserve the maternal antibody pool, but this has not yet been described. Protective antibodies are not repleted as with IVIg. No FcRn blocker has yet been tested in the pregnancy setting.
Peptidomimetics	A peptidomimetic is a small peptide designed to neutralize an antibody by mimicking its target. FISLE-412: a non-immunogenic peptidomimetic that neutralises anti-dsDNA antibodies involved in the pathogenesis of SLE; had success in preclinical trials and recent work has been done to reduce the structural complexity of the original saquinavir-derived FISLE-412 [83].	Specifically inhibits the pathogenic autoantibody Requires definition of the epitope targets of the pathogenic antibody, and drug design can be a challenge [82] The peptides may trigger the formation of immune complexes, which in turn may lead to (transient) inflammation, another risk factor for autism development. However, there is potential for design of a non-immunogenic molecule. No testing has occurred in the pregnancy setting.
Non-destructive monoclonal IgG	Antibodies with the same specificity as the pathogenic antibody are manufactured to lack effector functions, thus compete with the pathogenic antibody without causing disease. Potential use in treatment of FNAIT: Bakchoul et al. (2013) [88]: NGM-SZ21 inhibited PLT destruction induced by maternal anti-HPA-1alpha antibodies. Ghevaert et al. (2013) [89]: B2G1Δnab was confirmed in human volunteers as efficacious for treatment of FNAIT.	A less widely discussed approach but specifically described for treatment of maternal-autoantibody-mediated fetal disease Highly specific approach, likely with few adverse effects No transient immunosuppression Human and animal studies have demonstrated the safety of this approach to the foetus for certain antigen targets; however, safety and efficacy are dependent on the mechanism of pathogenesis of the autoantibody.
